# Gut Dysbiosis in Ocular Mucous Membrane Pemphigoid

**DOI:** 10.3389/fcimb.2022.780354

**Published:** 2022-04-14

**Authors:** Liying Low, Kusy Suleiman, Mohith Shamdas, Kerolos Bassilious, Natraj Poonit, Amanda E. Rossiter, Animesh Acharjee, Nicholas Loman, Philip I. Murray, Graham R. Wallace, Saaeha Rauz

**Affiliations:** ^1^ Academic Unit of Ophthalmology, Institute of Inflammation and Ageing, University of Birmingham, Birmingham, United Kingdom; ^2^ Birmingham and Midland Eye Centre, Sandwell and West Birmingham National Health Service (NHS) Trust, Birmingham, United Kingdom; ^3^ Institute for Microbiology and Infection, University of Birmingham, Birmingham, United Kingdom; ^4^ College of Medical and Dental Sciences, Institute of Cancer and Genomic Sciences, Centre for Computational Biology, University of Birmingham, Birmingham, United Kingdom; ^5^ Institute of Translational Medicine, University Hospitals Birmingham National Health Service (NHS), Foundation Trust, Birmingham, United Kingdom; ^6^ National Institute for Health Research (NIHR) Surgical Reconstruction and Microbiology Research Centre, University Hospital Birmingham, Birmingham, United Kingdom

**Keywords:** ocular mucous membrane pemphigoid (OcMMP), gut microbiome, inflammation, eye, 16S sequencing

## Abstract

Mucous Membrane Pemphigoid is an orphan multi-system autoimmune scarring disease involving mucosal sites, including the ocular surface (OcMMP) and gut. Loss of tolerance to epithelial basement membrane proteins and generation of autoreactive T cell and/or autoantibodies are central to the disease process. The gut microbiome plays a critical role in the development of the immune system. Alteration in the gut microbiome (gut dysbiosis) affects the generation of autoreactive T cells and B cell autoantibody repertoire in several autoimmune conditions. This study examines the relationship between gut microbiome diversity and ocular inflammation in patients with OcMMP by comparing OcMMP gut microbiome profiles with healthy controls. DNA was extracted from faecal samples (49 OcMMP patients, 40 healthy controls), amplified for the V4 region of the 16S rRNA gene and sequenced using Illumina Miseq platform. Sequencing reads were processed using the bioinformatics pipeline available in the mothur v.1.44.1 software. After adjusting for participant factors in the multivariable model (age, gender, BMI, diet, proton pump inhibitor use), OcMMP cohort was found to be associated with lower number of operational taxonomic units (OTUs) and Shannon Diversity Index when compared to healthy controls. Within the OcMMP cohort, the number of OTUs were found to be significantly correlated with both the bulbar conjunctival inflammation score (p=0.03) and the current use of systemic immunotherapy (p=0.02). The linear discriminant analysis effect size scores indicated that *Streptococcus* and *Lachnoclostridium* were enriched in OcMMP patients whilst *Oxalobacter, Clostridia uncultured genus-level group (UCG) 014, Christensenellaceae R-7 group* and butyrate-producing bacteria such as *Ruminococcus, Lachnospiraceae, Coprococcus, Roseburia, Oscillospiraceae UCG 003, 005, NK4A214 group* were enriched in healthy controls (Log10 LDA score < 2, FDR-adjusted p <0.05). In conclusion, OcMMP patients have gut dysbiosis correlating with bulbar conjunctival inflammation and the use of systemic immunotherapies. This provides a framework for future longitudinal deep phenotyping studies on the role of the gut microbiome in the pathogenesis of OcMMP.

## Introduction

Mucous membrane pemphigoid (MMP) is a rare, orphan, life-threatening autoimmune scarring disease, involving all mucosal sites, such as the ocular, oral, nasopharyngeal, anogenital, tracheal, oesophageal mucosa, and skin (Williams et al., 2011; Dart, 2017; Rashid et al., 2021). MMP with ocular involvement (OcMMP) is the commonest cause of cicatrising conjunctivitis in the United Kingdom, accounting for 61% of newly diagnosed cases, with an estimated minimum incidence of 0.8 per million population (Radford et al., 2012). Progressive autoimmune conjunctival fibrosis occurs both in inflamed eyes and in 50% of eyes that are clinically free from inflammation, leading to 20% of patients registered as blind (Williams et al., 2011). The pathogenesis of OcMMP involves a combination of both genetic and environmental factors leading to the loss of tolerance to conjunctival epithelial basement membrane proteins, and generation of autoreactive T cell and/or autoantibodies to basement membrane proteins that are central to the disease process (Dart, 2017). The mechanisms on how and where the T cells first become reactive to self-antigens in OcMMP is unknown.

There is mounting evidence that the gut microbiome plays a critical role in the development and maturation of the immune system and ocular inflammation (Kodati and Sen, 2019; Mendez et al., 2020; Moon et al., 2020; Scaglione et al., 2020; Napolitano et al., 2021). The gut microbiota affects the generation of autoreactive T cells and B cell autoantibody repertoire (Lee et al., 2011; Belkaid and Harrison, 2017; Li et al., 2020). Alteration in the gut microbiome (gut dysbiosis) through broad spectrum oral antibiotic ingestion has been associated with increased recruitment of effector T cells and ocular surface inflammation in a mouse model (C57BL/6 mice) of desiccating stress (De Paiva et al., 2016). Restoration of the gut microbiome through faecal transplant decreased the number of autoreactive CD4^+^IFN- γ^+^ T cells and reduced ocular surface inflammation (Wang et al., 2018). In a murine model of spontaneous uveitis (R161H mice), Caspi and associates showed that activation of retina-specific T cell receptor was dependent on the gut microbiota (Horai et al., 2015). Several small clinical studies have reported differences in the gut microbiota composition of Sjögren’s Syndrome (SS) patients with dry eyes compared to non-SS dry eye syndrome patients and healthy individuals, with reduction in gut microbial diversity correlating with ocular surface and systemic inflammation (De Paiva et al., 2016; Mendez et al., 2020; Moon et al., 2020). Elhusseiny and associates have recently reported an interesting case of a patient with refractory OcMMP and ulcerative colitis despite being on aggressive immunosuppressive therapy, who had long-term remission of ocular surface inflammation following colectomy (Elhusseiny et al., 2019). The gut microbiome can be shaped by the host genotype, in particular the genes encoding for the human leukocyte antigen (HLA) molecules which may confer genetic susceptibility to autoimmunity (Olivares et al., 2015). The loss of tolerance to basement membrane proteins is associated with enhanced susceptibility in OcMMP patients with the major histocompatibility complex (MHC) class II allele, HLA-DQB*0301 and HLA-DR4 (Zaltas et al., 1989; Delgado et al., 1996; Setterfield et al., 2001). However, in a familial case study of a pair of female monozygotic twins that share the same HLA haplotypes, only one twin developed OcMMP, whilst the other twin and her children were unaffected (Bhol et al., 1995). This suggests that pathogenesis of OcMMP could be multi-gene and associated with other environmental factors.

We examined whether differences in the gut microbiome were associated with disease manifestation in OcMMP. In this study, we show that gut microbiome dysbiosis in patients with OcMMP is characterised by lower numbers of operational taxonomic units (OTUs) and Shannon Diversity Index when compared to healthy controls and is linked to severity in bulbar conjunctival inflammation.

## Materials and Methods

### Participant Enrolment and Sample Collection

The study was approved by the London Bridge Research Ethics Committee [Systemic Gut Microbiota Driving Sight-threatening Inflammatory Ocular Disease (STUDIOUS), reference: 17/LO/0062, IRAS project ID: 140601)] and written informed consent was obtained from all participants. The research study was conducted in accordance with the World Medical Association’s Declaration of Helsinki.

Patients diagnosed with OcMMP attending the Ocular Surface Disease clinics at the Birmingham and Midland Eye Centre, Birmingham, United Kingdom were invited to participate in the study. The diagnosis of OcMMP was based on clinical findings typical of OcMMP [subepithelial fibrosis, loss of plica, symblepharon, limbitis, forniceal foreshortening, symblepharon formation with or without a history of intermittent or persistent conjunctival inflammation (acute or chronic)] after exclusion of other causes of cicatrising conjunctivitis, regardless of direct immunofluorescence biopsy results (Ong et al., 2018; Rashid et al., 2021). Direct immunofluorescence (DIF) biopsy result was deemed positive based on the presence of IgG, IgA and/or C3 deposits in the epithelial basement membrane zone (Chan et al., 2002) but a positive test was not essential for the diagnosis of OcMMP (Radford et al., 2012; Rashid et al., 2021).

A validated clinical assessment tool for cicatrising conjunctivitis (CCAT) was used to measure conjunctival inflammation (disease activity) in patients with OcMMP at time of recruitment to the study as outlined by Ong et al (Ong et al., 2020). The Cicatrising Conjunctivitis Assessment Tool scoresheet, inflammation grading photographs and guidelines for use document were available online (Cicatrising Conjunctivitis Assessment Tool - Scoresheet, Inflammation Grading Photographs and Guidelines for Use, 2021). Disease activity was based on the visible inflammation score based upon bulbar conjunctival hyperaemia grading scales, grading each quadrant on a 5-point (0–4) scale using the standardised grading scale panel of photographs (Cicatrising Conjunctivitis Assessment Tool - Scoresheet, Inflammation Grading Photographs and Guidelines for Use, 2021) [Maximum total of 16; Nil: 0 = 0%; Minimal: 1 – 4 (range 1 – 25%); Mild: 5 – 8 (range 26 – 50%); Moderate: 9 – 12 (range 51 – 75%); Severe: 13 – 16 (range 76 – 100%)] and Limbitis [max total of 4 scored as present or absent in each quadrant]. As there were no patients who had limbitis, all scores, for the purposes of the analyses were restricted to bulbar conjunctival inflammation scores [1-16 (1-100%)] in the worst eye (Cicatrising Conjunctivitis Assessment Tool - Scoresheet, Inflammation Grading Photographs and Guidelines for Use, 2021). Scarring was scored based on the extent of symblepharon, graded as absent (score of 0), if less than half horizontal involvement by symblepharon of horizontal fornix intercanthal distance (score of 1), if more than or equal to half horizontal involvement by symblepharon of horizontal fornix intercanthal distance (score of 2) (Ong et al., 2020). The morbidity score was based on corneal vascularisation and corneal opacity. Each peripheral corneal quadrant was scored as positive with a score of 1 for the involvement by vessels or opacity separately. For corneal vascularisation, a score of 1 was given to each peripheral quadrant involved, and if the central cornea was involved, a score of 1 was given, giving a maximum score of 5. For corneal opacification, a score of 1 was given to each peripheral quadrant involved, and an additional score of 5 if the central cornea was involved, giving a maximum score of 9. [Maximum total morbidity score of 14; score 1-14 (1-100%) in the worst eye] (Ong et al., 2020; Cicatrising Conjunctivitis Assessment Tool - Scoresheet, Inflammation Grading Photographs and Guidelines for Use, 2021).

Immunosuppressive treatment for OcMMP followed a “step-ladder” approach guided by disease activity as outlined by Rauz and associates (Rauz et al., 2005; Williams et al., 2011; Schmidt et al., 2021). OcMMP patients with minimal inflammation were treated with low dose oral tetracycline (doxycycline, 50-100mg once a day) for its anti-inflammatory properties (Dart, 2017) or dapsone (25-50mg twice a day). Patients with persistent disease, or those with mild to moderate bulbar conjunctival hyperaemic inflammation, either azathioprine (1 – 2.5mg/kg/day) or mycophenolate mofetil (500 – 1000 mg twice a day) was initiated. For patients with severe inflammation, either continuous oral cyclophosphamide (1 – 2 mg/kg/day) and adjuvant prednisolone (1 mg/kg/day) or pulsed oral or intravenous cyclophosphamide with intravenous methylprednisolone was delivered to induce rapid remission before step-down to less toxic therapy (Khan et al., 2013). For patients with refractory disease, intravenous anti-CD20 monoclonal antibody was employed (Saw et al., 2008; Williams et al., 2011; Schmidt et al., 2021).

Healthy adult volunteers were identified from visitors or staff working at the Birmingham and Midland Eye Centre, or from the University of Birmingham *1000 Elders project*, and invited to participate in the study. The *Birmingham 1000 Elders* is a cohort of healthy older adults above the age of 60 years involved in research at the University of Birmingham (courtesy of Professor Janet Lord) (The Birmingham 1000 Elders Group - University of Birmingham, 2021). Factors such as age and gender were used to match the OcMMP group as close as possible with the healthy controls. Body mass index (BMI) was higher in the OcMMP group, and it was difficult to obtain a ‘healthy control’ group with equally high BMI without introducing underlying metabolic dysfunction.

Exclusion criteria for both OcMMP and healthy controls were as follows: history of bowel surgery, inflammatory bowel syndrome, or systemic malignancy. Participants were provided with a faecal sample collection kit comprising clean nitrile gloves, sterile faecal sample collection container containing 97% ethanol, re-sealable plastic bag with absorbent pad, disposable paper collection sheet to cover over the toilet seat, an instruction leaflet and pre-paid postal package. Participants collected a single faecal sample produced at any time of day. Collection containers were placed in a pre-paid postal package and delivered *via* First class Royal Mail^®^ services at room temperature. Upon receipt of samples, aliquots were frozen at -80°C until DNA extraction. Participants were asked to complete a questionnaire indicating their dietary preferences - standard diet which includes intake of meat (red meat/poultry) and/or seafood or non-standard diet (vegetarian, vegan or other types of diet).

### DNA Extraction From Faecal Samples, 16S rRNA Gene Amplification, Illumina MiSeq Amplicon Sequencing

Faecal DNA was extracted using DNeasy PowerLyzer Powersoil Kit (Qiagen, Hilden, Germany) according to the manufacturer’s protocol. DNA yield was evaluated by Qubit fluorometer (Thermo Fisher Scientific, Waltham, MA, USA). The 16S rRNA V4 region was amplified by PCR and sequenced on the Illumina MiSeq platform (Illumina Inc, San Diego, California, USA) using the dual-indexed 2 x 150 bp paired end protocol modified from Kozich et al (Kozich et al., 2013). Briefly, 16S rRNA gene libraries were constructed from the genomic faecal DNA using primers to amplify the V4 region (250bp) using the primers (16Sf: GTGCCAGCMGCCGCGGTAA, 16Sr: GGACTACHVGGGTWTCTAAT). Each PCR reaction consisted of 0.5μl Phusion Polymerase (Thermo Fisher Scientific, Waltham, MA, USA), 1.5μl DMSO (Thermo Fisher Scientific, Waltham, MA, USA), 1μl each of V4 primer at 10μM, and 1μl DNA template (1ng). PCR conditions consisted of an initial 30 seconds at 98°C denaturation step, followed by 28 cycles of 10 seconds denaturation at 98°C, 30 seconds annealing cycle at 60°C, and 10 seconds extension cycle at 72°C, with final extension for 10 minutes at 72°C and kept at 4°C. Amplicon clean-up was done using 0.85x of Agencourt AMPure XP magnetic purification beads (Beckman Coulter, Indianapolis, USA). The concentration of the 16S library pools was determined using Qubit 3.0 Fluorometer (Thermo Fisher Scientific, Waltham, MA, USA) and the library pool was diluted to 2nM. Sequencing was performed on the Illumina Miseq platform using the Miseq Reagent Kit V2 500 cycle reagent kit (Illumina Inc, San Diego, California, USA). Negative (blank, no DNA template) and positive controls (aliquots of faecal sample from one healthy, ‘generous’ donor) were used for DNA extraction, 16S amplification and Illumina MiSeq sequencing.

### Bioinformatic Analysis and Statistics

Sequencing reads were processed using bioinformatics pipeline available in the mothur v.1.44.1 software (Schloss et al., 2009) – assembly of contigs between read pairs, alignment to reference region, trimming of sequence ends to the same alignment coordinates, preclustering to denoise sequences within each sample, screening for chimeras using UCHIME, classification against the SILVA reference files release 138 (Quast et al., 2013) using a naïve Bayesian classifier, split into groups at the level of order, and assignment to operational taxonomic units (OTUs) at a level of 3% dissimilarity. Alpha diversity was measured using by calculating the Shannon index and observed number of OTUs using mothur (Schloss et al., 2009). Principal coordinate analysis (PCoA) plots were created after calculating for distance matrices, demonstrating the distance between samples in two dimensions – the further the distances, the greater the difference in the microbiome composition. Comparisons were made using Yue and Clayton theta distances between samples – the larger Yue and Clayton theta distance, the more dissimilar the bacterial populations between the samples. The analysis of molecular variance (AMOVA) and homogeneity of molecular variance (HOMOVA) were conducted within mothur (Schloss et al., 2009). Samples were examined for differentially abundant bacterial taxa using linear discriminant analysis of effect size (LEfSe) within mothur (Segata et al., 2011) and adjusted for multiple hypothesis testing using the Benjamini and Hochberg method (R: The R Project for Statistical Computing, 2020). Continuous data were summarised using medians and interquartile ranges. Statistical analyses (Mann-Whitney, Spearman non-parametric correlation) were performed using GraphPad Prism v.8.0.0 (GraphPad Software, San Diego, California, USA) whilst linear regressions were performed using SPSS v.26.0 (IBM Corp., Armonk, NY) with p-value of less than 0.05 deemed to be indicative of significance throughout. To explore the relationship between patient factors and the number of OTUs, univariable linear regression models were initially produced for all factors of interest. All factors were then entered into a multivariable regression model, alongside the patient cohort (OcMMP *vs*. control), in order to identify factors that were independently associated with the numbers of OTUs. Multicollinearity was assessed using the variance inflation factor (VIF), with instances of VIF>5 noted, and VIF>10 taken to indicate serious multicollinearity warranting further investigation (Kim, 2019).

## Results

Faecal samples were collected from 49 OcMMP patients and 40 healthy volunteers. Participant characteristics and details of medications are shown in **Tables 1** and **2**. Notably 50% of patients required the use of oral tetracyclines and 77% required systemic immunosuppression either at some point of their disease course or currently. The demographics were comparable between the OcMMP and healthy cohorts, except that the OcMMP cohort had a higher BMI (p<0.001). A majority of the OcMMP patients had positive direct immunofluorescence (DIF) biopsy results (69%), with a median duration of disease of 33 months [Interquartile range (IQR): 13 – 98 months]. None of the OcMMP patients reported active extraocular symptoms.

**Table 1:** Demographics of participants.

	MMP (n=49)	HC (n=40)	p-Value
**Age (years)**	70 ± 11	71 ± 9	0.820
**Gender (female, %)**	31 (63%)	20 (50%)	0.282
**Ethnicity (White, %)**	47 (96%)	39 (98%)	1.000
**BMI (kg/m^2^)^§^**	28.7 (25.5-32.5)	23.6 (22.0-25.9)	**0.001**
**Proton pump inhibitor use**	5 (10%)	4 (10%)	1.000
**Diet (standard diet, %)^‡^**	43 (93%)	32 (82%)	0.175
**Duration of disease from diagnosis (months)**	33 (13-98)	N/A	–
**Positive immunofluorescence status**	34 (69%)	N/A	–
**Bulbar conjunctival hyperaemia score ≥25%^†^**	14 (29%)	N/A	–
**Tetracycline use**	24 (50%)		–
* Never*	25 (50%)	N/A	
* Previously, Now Stopped*	6 (14%)	N/A	
* Currently*	18 (36%)	N/A	
**Systemic immunotherapy use**	38 (78%)		–
* Never*	11 (22%)	N/A	
* Previously, Now Stopped*	10 (20%)	N/A	
* Currently*	28 (57%)	N/A	
			
**Observed number of OTUs**	225 ± 80	323 ± 87	**<0.001**
**Shannon Diversity Index**	2.92 ± 0.67	3.48 ± 0.69	**<0.001**

Data reported as median and interquartile range or mean and standard deviation, with p-values from Mann-Whitney U tests, or as n (%), with p-values from Fisher’s exact tests. BMI, body mass index; DIF, direct immunofluorescence. Bold p-values are significant at p<0.05.

^§^Data available for 83 participants.

^‡^Data available for 85 participants.

^†^Bulbar conjunctival inflammation score (in the worse eye) was assessed using the Cicatrising Conjunctivitis Clinical Assessment Tool.

N/A, Non-applicable.

**Table 2:** Details of medications/systemic immunotherapy of the 49 OcMMP patients (percentages are based on the total number of OcMMP patients recruited to the study, denominator = 49).

	Total number of patients who have required immunosuppression	Currently taking medication	Previously used, now stopped	Never used
**Azathioprine**	12 (24%)	6 (12%)	6 (12%)	37 (76%)
**Mycophenolate**	25 (51%)	14 (29%)	11 (22%)	24 (49%)
**Cyclophosphamide**	12 (24%)	2 (4%)	10 (20%)	37 (76%)
**Biologics**	2 (4%)	0 (0%)	2 (4%)	47 (96%)
**Systemic steroids**	23 (47%)	7 (14%)	16 (32%)	26 (53%)
**Dapsone**	6 (12%)	2 (4%)	4 (8%)	43 (88%)

A median of 110,134 sequences were generated per sample, with an IQR of 74,230 to 123,647 sequences. Samples were rarefied to 25,000 sequences. Three samples had sequences lower than 25,000 and were therefore excluded from the bioinformatic analyses (two healthy controls, and one OcMMP). The average coverage was 99.7%.

### Differences in Alpha-Diversity Between OcMMP and Healthy Controls

Measures of alpha-diversity (observed OTUs and Shannon Diversity Index) were assessed to determine differences in the microbial diversity within each sample. After adjusting for participant factors in the multivariable model (age, gender, BMI, diet, proton pump inhibitor use), OcMMP cohort was found to be significantly independently associated with lower number of OTUs, with adjusted number of OTUs of 115 (95% CI: 73-157, p<0.001) and lower Shannon Diversity Index, with an adjusted Shannon Diversity Index 0.70 (95% CI: 0.35-1.04, p<0.001) lower than in healthy controls (**Tables 3** and **4**).

**Table 3:** Linear regression models of number of OTUs in OcMMP vs. healthy controls.

Variables	Univariable Models		Multivariable Model	
	Coefficient (95% CI)	p-Value	Coefficient (95% CI)	p-Value
**Cohort (OcMMP)**	-98.20 (-133.96, -62.44)	**<0.001**	-111.91 (-153.63, -70.20)	**<0.001**
**Age (per decade)**	11.74 (-8.77, 32.34)	0.258	13.22 (-6.76, 33.21)	0.156
**Gender (Female)**	-5.31 (-47.38, 36.75)	0.802	15.99 (-25.61, 57.58)	0.295
**BMI (per 5kg/m^2^)**	-13.45 (-31.15, 4.26)	0.134	-5.58 (-23.29, 12.13)	0.685
**Diet (Standard diet)**	2.47 (-63.57, 68.52)	0.941	59.37 (-4.98, 123.73)	0.010
**PPI use (Yes)**	-16.74 (-87.82, 54.34)	0.641	5.54 (-59.39, 70.47)	0.994

Results are from linear regression models. The coefficient represents the increase in the number of OTUs per the stated number of units increase for continuous variables, or for the stated category relative to the reference category for nominal variables. Bold p-values are significant at p<0.05. The number of participants in each group of the categorical variables are as per **Table 1**.

**Table 4:** Linear regression models of Shannon Diversity Index in OcMMP vs. healthy controls.

Variables	Univariable models		Multivariable models	
	Coefficient (95% CI)	p-Value	Coefficient (95% CI)	p-Value
**Cohort (OcMMP)**	-0.57 (-0.86, -0.27)	**<0.001**	-0.68 (-1.03, -0.33)	**<0.001**
**Age (per decade)**	0.11 (-0.05, 0.26)	0.182	0.11 (-0.06, 0.27)	0.198
**Gender (Female)**	0.02 (-0.31, 0.34)	0.921	0.19 (-0.15, 0.54)	0.266
**BMI (per 5kg/m^2^)**	-0.05 (-0.18, 0.10)	0.537	0.01 (-0.14, 0.16)	0.919
**Diet (Standard diet)**	0.02 (-0.49, 0.52)	0.945	0.42 (-0.11, 0.96)	0.120
**PPI use (Yes)**	0.16 (-0.39, 0.70)	0.564	0.31 (-0.23, 0.85)	0.255

Results are from linear regression models. The coefficient represents the increase in the number of OTUs per the stated number of units increase for continuous variables, or for the stated category relative to the reference category for nominal variables. Bold p-values are significant at p<0.05. The number of participants in each group of the categorical variables are as per **Table 1**.

**Figure 1:**
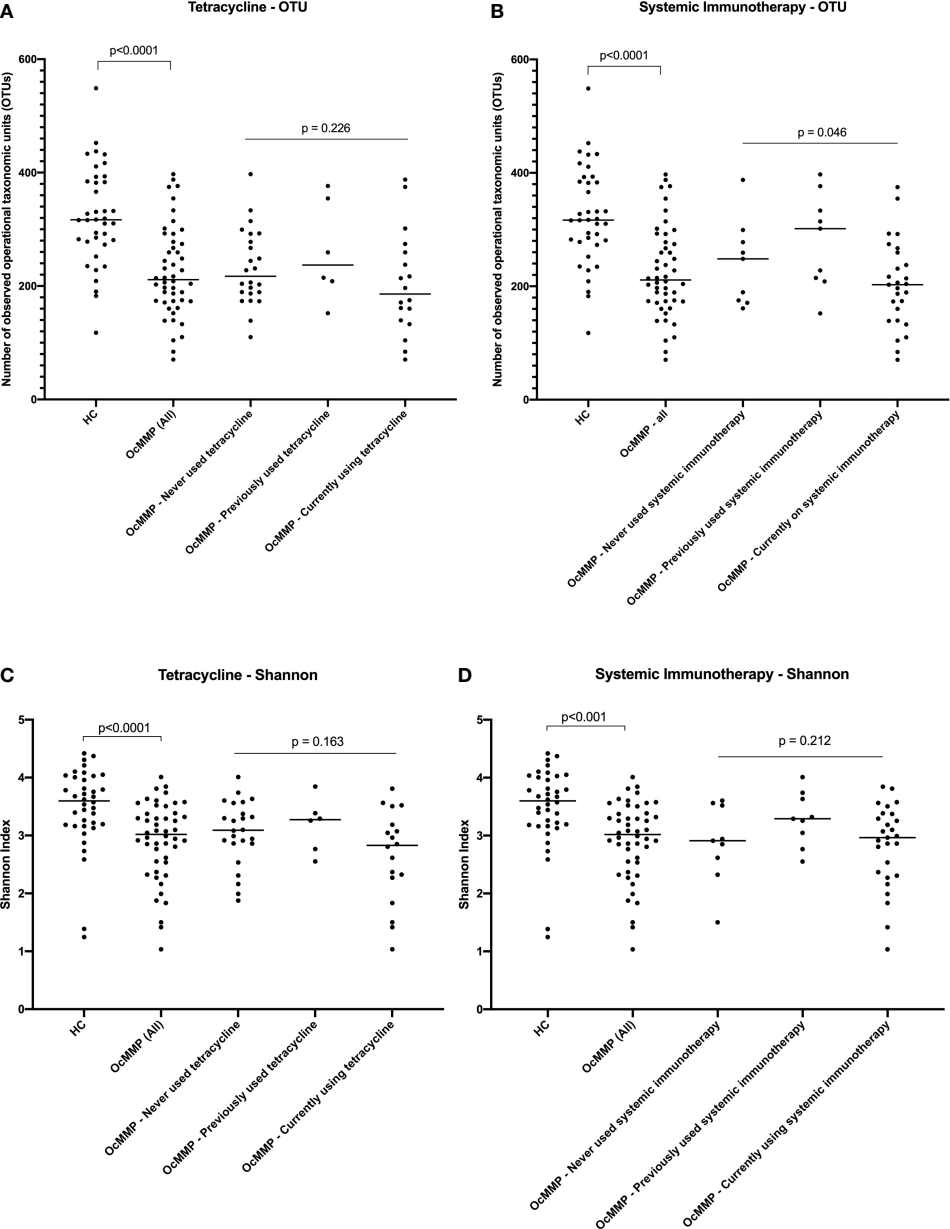
Comparisons in alpha diversity between healthy volunteers and OcMMP. Comparisons between the HC and OcMMP groups were initially performed using Mann-Whitney U tests. The OcMMP group was then divided into three subgroups, based on systemic immunotherapy usage, and comparisons between these subgroups were performed using Kruskal-Wallis tests. Comparisons in observed operational taxonomic units (OTUs) and Shannon Index between healthy controls and OcMMP, with subsets of OcMMP patients in relation to their use of low-dose oral tetracycline **(A, C)**, and use of systemic immunotherapy **(B, D)**. Patients with OcMMP had significantly lower number of OTUs and Shannon Index compared to healthy controls. No significant differences in number of OTUs or Shannon Index were detected between OcMMP patients who had never used, previously used or currently on long-term, sub antimicrobial dose of oral tetracycline, known to exert anti-inflammatory effects through its matrix metalloproteinase inhibitor properties **(A, C)**. Patients who were currently on systemic immunomodulatory therapy had lower observed OTUs compared to those who had never used or had previously used systemic immunotherapy, however, there was no significant difference in the Shannon Index for these patients **(B, D)**.

To determine if systemic treatment or clinical disease in OcMMP patients could be contributing to the alpha-diversity differences seen in the gut microbiome, observed OTUs and Shannon index parameters were assessed within the OcMMP cohort. No significant alpha-diversity differences were detected between OcMMP patients who had never used, previously used or currently on long-term, sub antimicrobial dose of oral tetracycline, known to exert anti-inflammatory effects through its matrix metalloproteinase inhibitor properties (**Figures 1A, C**). Patients who were currently on systemic immunomodulatory therapy had lower observed OTUs compared to those who had never used or had previously used systemic immunotherapy, however, there was no significant difference in the Shannon Index for these patients (**Figures 1B, D**). No significant alpha-diversity differences, as quantified by the number of OTUs (p=0.88) or Shannon Index (p=0.47), were detected between OcMMP patients who had never used, previously used or were currently using dapsone.

**Figure 2:**
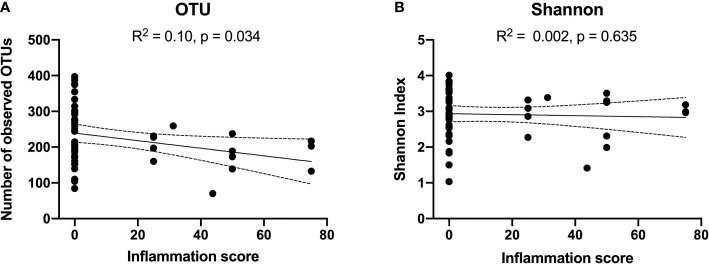
Correlations between alpha diversity [OTUs in Panel **(A)**, and Shannon Index in Panel **(B)**] and Cicatrising Conjunctivitis Clinical Assessment Tool (CCAT) score of bulbar conjunctival hyperaemia defined as ‘inflammation’ in the worse eye. A higher clinical inflammation score represents a more active clinical disease. Reduced number of observed OTUs were correlated with extent of bulbar conjunctival inflammation (R2: 0.1, p= 0.03). However, there was no significant correlation between Shannon Index and inflammation. OTUs, operational taxonomic units.

Reduced number of observed OTUs were weakly correlated with bulbar conjunctival inflammation (R^2^: 0.1, p= 0.03). However, there was no significant correlation between Shannon Index and inflammation (**Figures 2A, B**). No significant correlation was detected between alpha-diversity measures and the cicatrising conjunctivitis clinical assessment scores of scarring or morbidity. Alpha-diversity was not significantly associated with duration of disease or DIF biopsy results.

Associations between factors and alpha-diversity (number of OTUs) were assessed within the OcMMP cohort. On univariable analysis, the number of OTUs was found to be significantly correlated with both the ocular inflammation score (p=0.03) and the current use of systemic immunotherapy (p=0.02). However, on multivariable analysis, which additionally adjusted for age, gender, ethnicity, BMI, diet, proton pump inhibitor use, immunofluorescence status, tetracycline, dapsone or systemic immunosuppression use, none of the factors considered were identified as significant independent predictors of the number of OTUs, including bulbar conjunctival hyperaemia score (p=0.21) and the current use of systemic immunotherapy (p=0.11) [**Table 5**]. There was significant correlation between the bulbar conjunctival hyperaemia score and current use of systemic immunotherapy (Spearman 0.36, p = 0.01).

**Table 5:** Linear regression models of number of OTUs and ocular inflammation score.

Variables	Univariable model		Multivariable model	
	Coefficient (95% CI)	p-Value	Coefficient (95% CI)	p-Value
Bulbar Conjunctival Hyperaemia score	-1.06 (-2.02, -0.11)	**0.03**	-0.96 (-2.37, 0.45)	0.17
Age (per decade)	20.02 (-1.81, 41.85)	0.07	19.54 (-6.68, 45.76)	0.14
Gender (Female)	23.65 (-24.75, 72.05)	0.33	12.39 (-37.76, 62.53)	0.62
Ethnicity (White)	-96.11 (-209.63, 17.41)	0.10	-74.55 (-158.08, 8.98)	0.11
BMI (per 5kg/m^2^)	-1.68 (-22.80, 19.44)	0.87	2.25 (-28.20, 23.69)	0.86
Diet (Standard diet)	12.72 (-84.61, 110.05)	0.79	42.68 (-68.59, 153.96)	0.44
PPI use (Yes)	-33.39 (-109.32, 42.53)	0.38	12.4 (-86.76, 111.56)	0.80
Duration of disease from time of diagnosis (per year)	4.52 (-0.94, 9.98)	0.10	-0.66 (-8.28, 6.96)	0.86
Immunofluorescence status (Positive)	17.58 (-32.61, 67.78)	0.48	-1.58 (-53.65, 56.81)	0.95
Tetracycline use (current)	-34.52 (-81.74, 12.70)	0.15	-39.56 (-89.58, 10.46)	0.12
Dapsone use (current)	-25.39 (-142.20, 91.42)	0.66	-50.08 (-174.55, 74.40)	0.41
Systemic immunotherapy use (current)	-56.43 (-101.23, -11.63)	**0.02**	-43.21 (-99.81, 13.40)	0.13

Results are from linear regression models. The coefficient represents the increase in the number of OTUs per the stated number of units increase for continuous variables, or for the stated category relative to the reference category for nominal variables. Bold p-values are significant at p<0.05.

### Differences in Beta Diversity Between OcMMP and Healthy Controls

The differences in overall microbial community structure between OcMMP and healthy controls were determined by using Yue and Clayton theta distances. There were significant differences in the clustering within the OcMMP and healthy control ordinations (analysis of molecular variance (AMOVA) F statistics (Fs): 2.362, p < 0.001). There was no significant difference in the variation (homogeneity of molecular variance (HOMOVA) B value: 0.026, p = 0.239). No significant differences were detected in the clustering within OcMMP patients based on DIF biopsy results, treatment with tetracycline or systemic immunosuppression.

### Compositional Differences in Gut Microbiome of OcMMP and Healthy Controls

**Figure 3:**
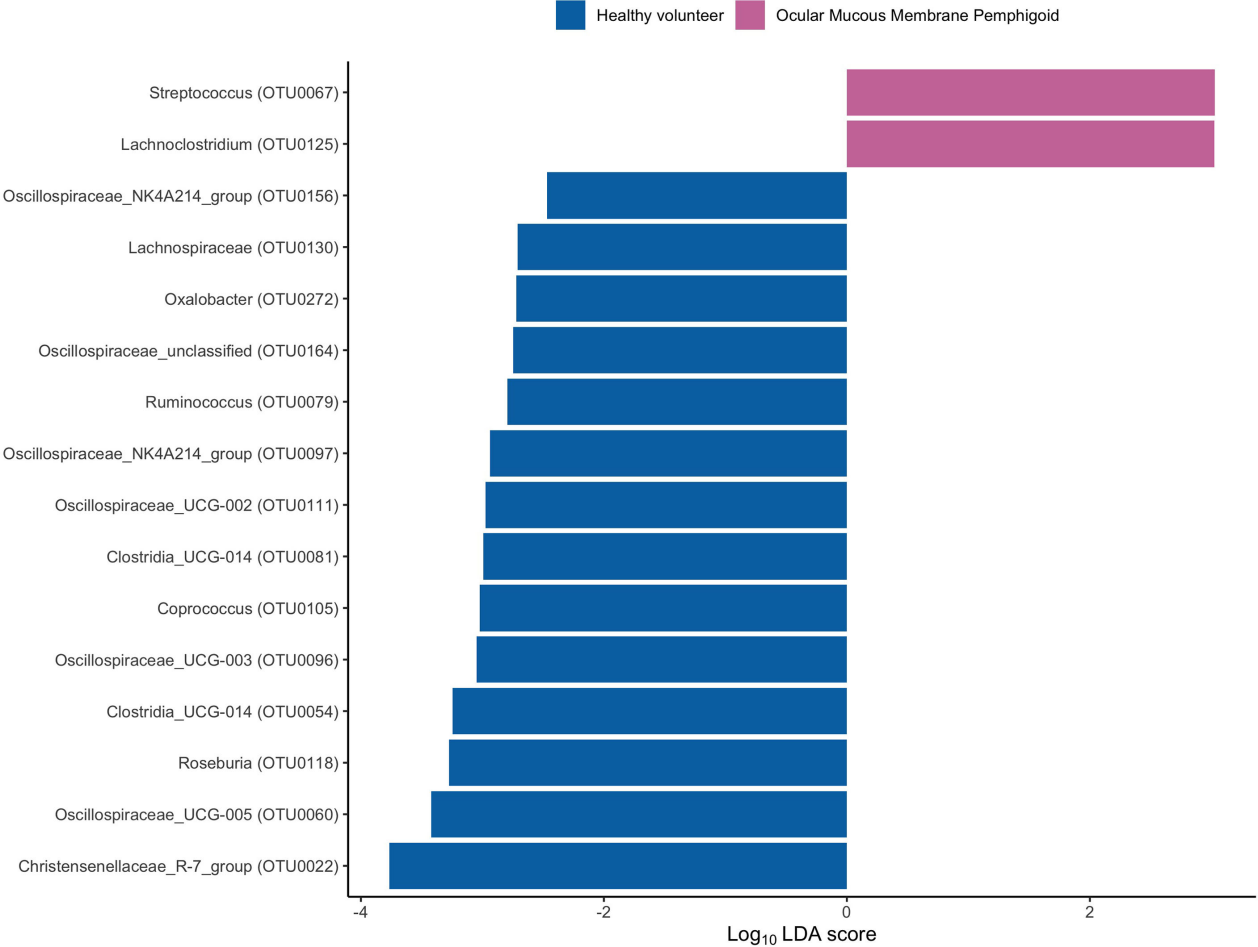
Linear discriminant analysis (LDA) effect size (LEfSe) of significantly enriched bacterial taxa in OcMMP and healthy volunteers.

**Figure 4:**
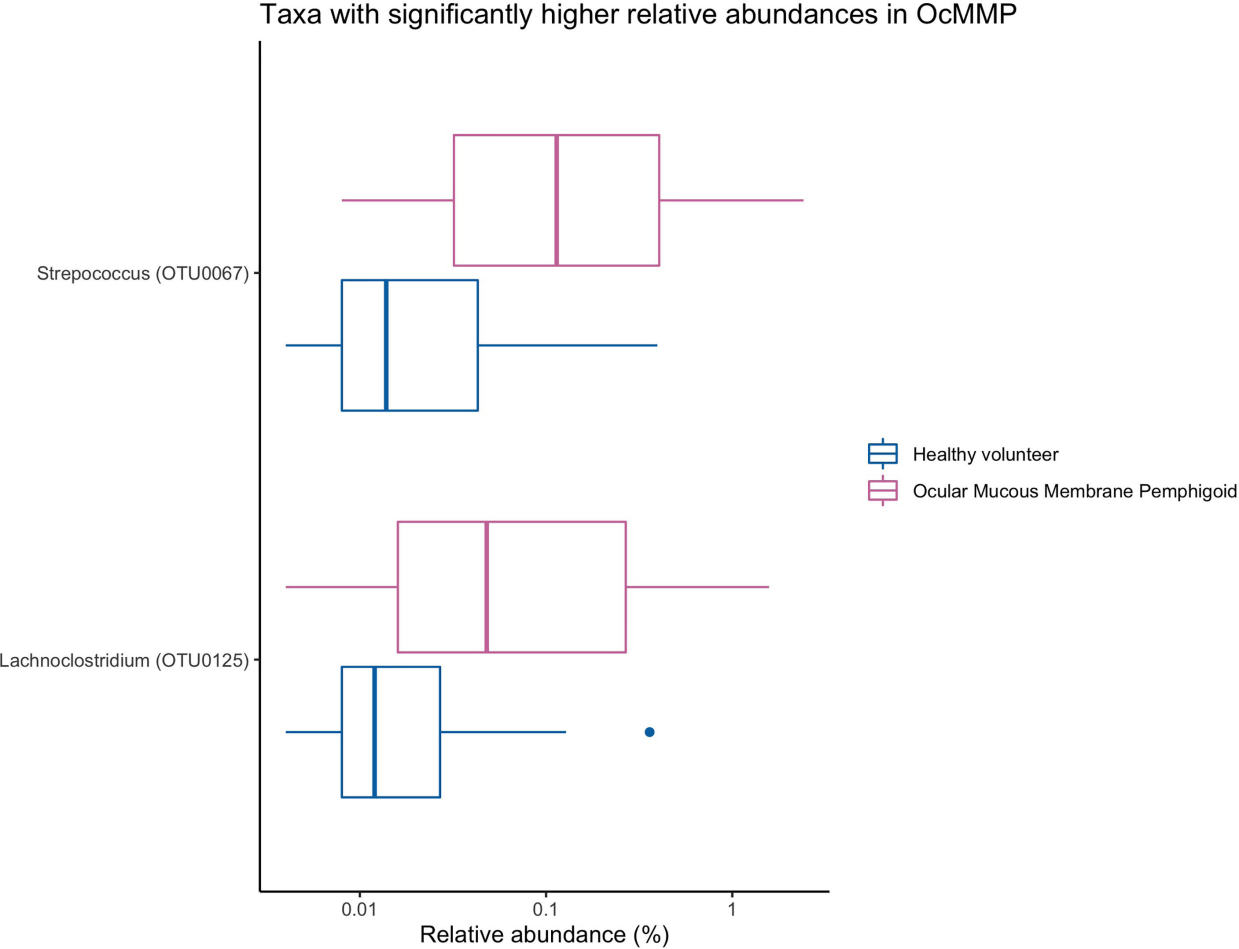
Comparisons of the relative abundances of bacterial taxa that were significantly enriched in OcMMP. *Streptococcus* and *Lachnoclostridium* were significantly enriched in OcMMP patients.

**Figure 5:**
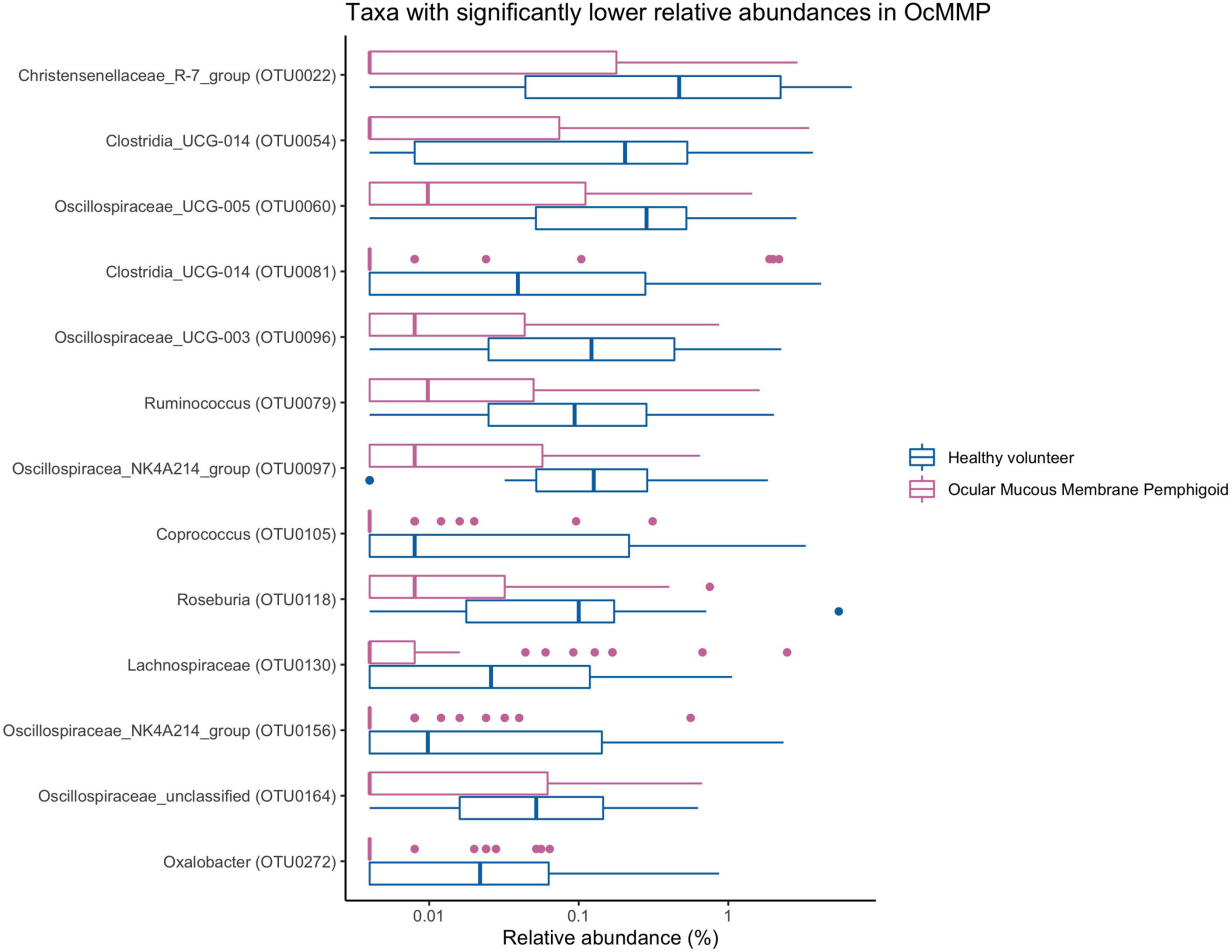
Comparisons of the abundances of bacterial taxa that are significantly enriched in healthy volunteers. *Oxalobacter, Clostridia uncultured genus-level group (UCG) 014, Christensenellaceae* R-7 group and butyrate-producing bacteria such as *Ruminococcus, Lachnospiraceae, Coprococcus, Roseburia, Clostridia uncultured genus-level group (UCG)-014, Oscillospiraceae UCG-003, 005, NK4A214 group* were enriched in healthy controls.

To assess the differences in microbial taxonomic abundances between OcMMP and healthy controls, I-assigned sequence reads were compared at various levels from phylum to genus. At the phylum level, the gut microbiome of both groups was dominated by Firmicutes and Bacteroidota/Bacteroides followed by Proteobacteria, Actinobacteriota and Desulfobacterota. The gut microbial dysbiosis between OcMMP and healthy controls were further analysed by linear discriminant analysis (LDA) effect size (LEfSe) method and revealed significant differences in the abundances of 16 bacterial taxa (**Figure 3**). The LDA scores indicated that *Streptococcus* and *Lachnoclostridium* were enriched in OcMMP patients whilst *Oxalobacter*, *Clostridia uncultured genus-level group (UCG) 014*, *Christensenellaceae R-7 group* and butyrate-producing bacteria such as *Ruminococcus*, *Lachnospiraceae*, *Coprococcus*, *Roseburia, Clostridia uncultured genus-level group (UCG)-014, Oscillospiraceae UCG-003, 005, NK4A214* group were enriched in healthy controls (Log_10_ LDA score > 2, FDR-adjusted p <0.05) (**Figures 4**, **5**).

## Discussion

This study is the first to compare the gut microbiome profile between patients with OcMMP and healthy controls using 16S rRNA gene sequencing. Patients with OcMMP were found to have lower alpha-diversity and altered gut bacterial composition compared with healthy controls. The reduction in the number of observed OTUs in the gut microbiome was associated with ocular surface inflammation scores using the CCAT and the use of systemic immunotherapy. There were no differences in the gut microbiome profile of OcMMP patients with DIF biopsy positive compared to those with DIF biopsy negative results. Taken together, these results suggest that OcMMP is associated with gut dysbiosis and correlates with bulbar conjunctival inflammation.

In this study, patients with OcMMP were observed to have lower alpha-diversity compared to healthy volunteers, in concordance with previously reported mouse model and human cohort studies in Sjögren’s Syndrome and uveitis (De Paiva et al., 2016; Kalyana Chakravarthy et al., 2018). Several bacterial taxa identified as being altered in OcMMP in this study have been associated with autoimmunity. The gut microbiome in patients with OcMMP were enriched for the genera of *Streptococcus* and *Lachnoclostridium*. Chakravarthy and associates performed 16S rRNA sequencing on the V3-V4 region of faecal samples from 13 patients with uveitis and 13 healthy controls in a small south Indian population, and have shown increased abundance of *Streptococcus*, and depletion of *Lachnospiraceae*, *Ruminococcaceae* in their uveitis patient cohort (Kalyana Chakravarthy et al., 2018). Enrichment of *Streptococcus* in the gut microbiome has also been reported in Sjögren’s Syndrome patients (De Paiva et al., 2016). The relative and absolute levels of *Streptococcus* spp in the gut microbiome correlated with higher osteoarthritis-related knee pain in a large, population-based Rotterdam cohort of over 1427 participants and was further validated in an independent cohort of 867 participants (Boer et al., 2019). Although unable to establish causality, the authors postulated that the metabolites or membrane vesicles containing endotoxin produced by the gastrointestinal luminal *Streptococcus* spp. may be responsible in eliciting extra-intestinal inflammation in the synovial lining (Boer et al., 2019). Given that the relative abundances of each taxa that were significantly altered in the OcMMP patient cohort accounted for less than 10% of the total gut microbiome, it is likely that a group of microbes sharing common metabolic or functional profiles, rather than a single predominant microbe, is associated with OcMMP.

Patients with OcMMP were also found to have decreased relative abundances of butyrate-producing bacteria such as *Ruminococcus*, *Lachnospiraceae*, *Oscillospiraceae*, *Coprococcus*, *Roseburia*, similar to previous gut microbiome studies in patients with pemphigus vulgaris, Sjögren’s syndrome, Behçet’s disease and uveitis (Consolandi et al., 2015; Kalyana Chakravarthy et al., 2018; Huang et al., 2019; Cano-Ortiz et al., 2020; Mendez et al., 2020). Butyrate, a short-chain fatty acid by-product of the large intestinal microbial fermentation, has been shown to induce and regulate the differentiation of T regulatory cells (Furusawa et al., 2013). Preliminary data by Hernandez and associates confirmed the presence and expression of butyrate receptors (G protein-coupled receptors 41, 43 and 109B) and sodium-coupled monocarboxylate transporter 1 (an electrogenic sodium- and chloride-dependent sodium-coupled solute transporter for butyrate) on the corneal and conjunctival epithelial cells (Hernandez et al., 2019). Treatment with butyrate blunted inflammatory responses in conjunctival and corneal epithelial cells, and bone marrow dendritic cells *ex-vivo*, suggesting a potential role of butyrate in modulating the ocular surface homeostasis (Hernandez et al., 2019). The gut microbiome is capable of modulating the host response to immunotherapies (Shui et al., 2020). Using metagenomic sequencing, Gopalakrishnan and associates reported an increase in the relative abundance of *Ruminococcaceae* and *Clostridales* in faecal samples of melanoma patients who responded well to anti-programmed death protein-1 therapy compared to those who did not (Gopalakrishnan et al., 2018a). Further functional *in-vivo* studies were carried out in germ-free mice showed that inoculation of mice with the identified bacteria, enhanced therapeutic effects of the anti-PD-1 therapy (Gopalakrishnan et al., 2018a). In another study conducted by Daillere and associates, oral administration of *Lactobacillus* and *Enterococcus* improved efficacy of cyclophosphamide in antibiotics-treated mice (Daillère et al., 2016). Additionally, memory Th1 immune responses to *Enterococcus hirae* and *Barnesiella intestinihominis* was associated with prolonged progression-free survival in 38 advanced lung and ovarian cancer patients receiving chemotherapy (Daillère et al., 2016). The gut microbiome has also been targeted for treatment of other autoimmune disorders, such as the use of *Lactobacillus casei* probiotic as an adjuvant therapy for rheumatoid arthritis (Ferro et al., 2021). Therefore, the modulation of the gut microbiome or its metabolites (e.g. short chain fatty acids) could potentially help in treatment of OcMMP, by improving the efficacy and reducing the side-effects of immunosuppressive treatments, as well as prolonging remission in patients with OcMMP.

In this study, gut dysbiosis was correlated with bulbar conjunctival inflammation in OcMMP, suggesting the gut microbiome could be driver of inflammation. It is also plausible that patients with more severe conjunctival inflammation could either had treatment with more aggressive systemic immunotherapies or had subclinical multi-systemic mucosal inflammation involving the intestinal system, thus affecting their gut microbiome. The lack of statistical significance of ocular inflammation and current systemic immunosuppression use in the multivariable model may be partly a consequence of multicollinearity given that there was a significant correlation between those two factors. A pros"pective multi-disciplinary systemic screening study undertaken by Ong and associates showed that a high proportion of OcMMP patients had asymptomatic disease at extra-ocular sites, including the gastrointestinal tract (Ong et al., 2018). Seminal work from de Paiva and associates demonstrated that primary Sjögren’s syndrome patients with the most severe dry eye disease had the lowest diversity in the gut microbiome (De Paiva et al., 2016). These data were further confirmed by Moon and colleagues (Moon et al., 2020). de Paiva and associates had also analysed the ocular surface microbiome in these patients and was not significantly different from healthy controls (De Paiva et al., 2016). This could be partly explained by the paucibacterial nature of the ocular surface secondary to the innate antimicrobial compounds found in tears and mechanical elimination through the blinking reflex or the lack of technical methods to sufficiently collect, extract and sequence the ocular surface microbiome (Doan et al., 2016).

There was no observable difference in the gut microbiome profile of patients with DIF biopsy positive and negative results. Given that DIF biopsy positivity requires the presence of circulating autoantibodies, it is likely that non-autoantibody driven components of inflammation, such as autoreactive T cells, could either contribute to or be affected by gut dysbiosis (Dart, 2017). We did not observe any significant correlation between BMI and gut microbiome profile in our study. Previous studies reporting changes in the gut microbiome in obesity and metabolic syndrome were comparing the profiles between obese (defined as BMI above 30 kg/m^2^) and non-obese individuals, whilst the median BMI in our patient cohort was within the ‘overweight’ category (Turnbaugh et al., 2006; Borgo et al., 2018; Gao et al., 2018; Boer et al., 2019; Defining Adult Overweight & Obesity | Overweight & Obesity | CDC, 2021). Study of 551 participants by Gao and associates showed that there was low among-group beta-diversity dissimilarities in the four groups of BMI (underweight, normal, overweight and obese) and that the changes in the gut microbiome composition was largely influenced by gender (Gao et al., 2018). Furthermore, meta-analysis of data from 10 studies performed by Sze and associates did not identiharacterizatble differences in the gut microbial composition and BMI (Sze and Schloss, 2016).

The link between the gut-eye axis remains unclear. Alterations in the gut microbiome could play a role in the pathogenesis of OcMMP or the immune responses in OcMMP pathophysiology on the mucosal surface could be driving the changes in the gut microbiome profile in OcMMP. Another potential confounder is the effect of treatment with systemic low-dose tetracycline and/or systemic immunosuppression on the gut microbiome in patients with OcMMP. There were no significant differences in the number of observed OTUs between patients who had never taken, previously used or currently using sub antimicrobial dose of tetracycline or dapsone. The tetracycline (doxycycline) maintenance dose, used primarily for its matrix metalloproteinase inhibitor (anti-inflammatory) properties, in this OcMMP patient cohort was 50mg daily, which is much lower than the usual 200mg – 100mg dose required for treating acute infections (BNF: British National Formulary - NICE). Similarly, the dapsone is used primarily for its anti-inflammatory capacity to suppress neutrophil superoxide production (BNF: British National Formulary - NICE; Suda et al., 2005; Wozel and Blasum, 2014). Antibiotics, even at sub-therapeutic doses, can cause gut dysbiosis by inducing blooms of certain strains of bacteria and depletion of other bacterial species (Elvers et al., 2020). Although the human gut microbiome is resilient, the rate of recovery or regrowth of the gut microbiome following antibiotics exposure is still unknown (Elvers et al., 2020). Palleja and associates showed that there is a long-lasting imprint in the gut microbiome following short-term antibiotic use (4-day intervention), with undetectable/loss of 9 common bacterial species (including butyrate producers) even after 180 days of stopping treatment (Palleja et al., 2018). This is further corroborated by evidence from a systematic review of 31 studies, involving 1068 participants, which observed that antibiotics, including doxycycline, can have a persistent effect on the gut microbiome – with marked decrease in Bifidobacterium (Elvers et al., 2020). The cumulative effect on the gut microbiome from long-term use of tetracycline or dapsone, bona-fide bacteriostatic antibiotics, at sub-therapeutic dose is still unclear. Studies have shown that antibiotic bacteriostatic/bactericidal activity is dose-dependent and that the gut microbiome is a reservoir for antibiotic-resistance genes (Doan et al., 2020; Maier et al., 2020).

There was significantly greater diversity (observed OTUs) in the gut microbiome of patients who had never been on systemic immunotherapy compared to those currently on treatment. This suggests that systemic inflammation influences the gut microbiome and those individuals who do not require immunotherapy may have less severe disease. Immunosuppressive therapies have been shown to change the gut microbial composition (Maier et al., 2018; Bhat et al., 2020), and in turn the gut microbiota may affect response to immunotherapy by modulating the drug metabolism or effectiveness of lymphocyte function (Gopalakrishnan et al., 2018b; Matson et al., 2018). Given the correlation between systemic immunotherapy use and ocular inflammation score, it is difficult to isolate the effects of these two factors in the current study. The fundamental principle in performing multivariate analyses to assess taxonomic variation is that sampling should be representative of the community of interest. Whilst larger sample sizes are ideal, Forcino and associates estimate that a conservative smaller sample size of 58 individuals is sufficient to produce statistically robust results using multivariate statistical analyses (Forcino et al., 2015). OcMMP is a rare, orphan disease with approximately 1600 patients in the UK and incidence of 0.8 per million population (Williams et al., 2011). Therefore, the patient sample size in our study constitutes approximately 3% of the UK cohort. A global multicentre study would ideally be required to give a sufficient sample size to perform either matched-cases or subgroup analyses to interrogate the effects between medications, host genotype and other environmental factors. Inclusion of another group of patients on immunosuppression for other conditions (i.e. disease control) may in theory enable treatment-based comparisons of the gut microbiome profile, but it is very difficult to source and match patients on similar immunosuppressive therapies (type of treatment, threshold for starting or switching treatment, duration, dose, disease response to treatment, and combination with other drugs). It would also be difficult to ascertain if changes in the gut microbiome were affected by the treatment or through the disease process itself (i.e., systemic *vs*. local gut inflammation). There are large intra- and interhost variations in the gut microbiome even amongst healthy individuals, as described by Vujkovic-Cvijin and associates (Vujkovic-Cvijin et al., 2020).

Using 16S rRNA gene sequencing, the current study is the first to characterise the gut microbiome in OcMMP, a very rare and orphan disease, in a relatively substantial number of patients (n=49) and correlating gut dysbiosis with clinical disease. There are several limitations to this study. This is a cross-sectional, observational study, therefore the distinction between causation or effect is inconclusive. Medications that are used in the treatment of OcMMP may have impact on the gut microbiome. Future longitudinal studies with larger patient cohort of varying stages of clinical activity and morbidity would be required to investigate the relationship between the changes in the gut microbiome and clinical parameters, along with treatment-associated gut microbiome alterations. The gut microbiome was profiled using 16S rRNA gene sequencing of faecal samples. Although 16S sequencing provides useful bacterial taxonomic information, metagenomic, metatranscriptomic sequencing and metabolic profiling of both the gut (representative of the luminal microbiota) and mucosal samples along with host inflammatory markers may reveal changes in the gut bacterial, viral, and fungi microbiome in relation to the host genetics and inflammatory status. There might be other lifestyle and environmental confounding factors that have not been accounted for in this study, such as alcohol intake, smoking status, and quality of bowel movement. This study likely reflects the current real-world challenges in determining the gut microbiome profile in a very rare disease cohort with complex clinical heterogeneity.

In summary, this study shows that OcMMP patients have gut dysbiosis and alterations in the gut microbiome correlated with disease activity. These preliminary correlative data will provide the framework for future deep phenotyping and integration with multiomic datasets, such as metatranscriptonomic, host genetics and metabolomics, and causative studies on the role of the gut microbiome in OcMMP and correlating with ocular surface microbiota as potential drivers of activity and damage components of disease. Modulation of the gut microbiome could potentially improve the efficacy and reduce the side-effects of immunosuppressive treatments, as well as prolonging remission (Gopalakrishnan et al., 2018a; Gopalakrishnan et al., 2018b; Moon et al., 2020). A larger international multi-centre study involving patients with ocular, oral, dermatological involvement, and followed longitudinally is required to address the relationship between the clinical phenotype of the disease and the gut dysbiosis. Further work on investigating the prognostic utility of gut microbiome profile in OcMMP and improving the characterisation of the ocular surface microbiome is required to provide insights into the gut-eye inflammatory axis in disease and health.

## Data Availability Statement

The datasets presented in this study can be found in online repositories. The names of the repository/repositories and accession number(s) can be found below: https://www.ebi.ac.uk/ena, PRJEB47631.

## Ethics Statement

The studies involving human participants were reviewed and approved by London Bridge Research Ethics Committee (Systemic Gut Microbiota Driving Sight-threatening Inflammatory Ocular Disease (STUDIOUS), reference: 17/LO/0062, IRAS project ID: 140601). The patients/participants provided their written informed consent to participate in this study.

## Author Contributions

LL, GW, PM, and NL contributed to conception and design of the study. LL, KS, and AR performed the laboratory work. LL, MS, and KS recruited participants to the study. LL performed the statistical analysis. LL wrote the first draft of the manuscript. AA contributed to bioinformatics analysis. All authors contributed to manuscript revision, read, and approved the submitted version.

## Funding

LL is funded through a Fight for Sight Clinical Research Fellowship (Ref 1840/1841) and a National Institute for Health Research (NIHR) Clinical Lectureship.

## Conflict of Interest

The authors declare that the research was conducted in the absence of any commercial or financial relationships that could be construed as a potential conflict of interest.

## Publisher’s Note

All claims expressed in this article are solely those of the authors and do not necessarily represent those of their affiliated organizations, or those of the publisher, the editors and the reviewers. Any product that may be evaluated in this article, or claim that may be made by its manufacturer, is not guaranteed or endorsed by the publisher.
